# Human β-Defensin 3 Inhibits *Porphyromonas Gingivalis* Lipopolysaccharide-Induced Oxidative and Inflammatory Responses of Microglia by Suppression of Cathepsins B and L

**DOI:** 10.3390/ijms232315099

**Published:** 2022-12-01

**Authors:** Erika Inoue, Shiyo Minatozaki, Yui Katsuta, Saori Nonaka, Hiroshi Nakanishi

**Affiliations:** 1 Faculty of Pharmacy, Yasuda Women’s University, Hiroshima 731-0153, Japan; 2 Department of Pharmacology, Faculty of Pharmacy, Yasuda Women’s University, Hiroshima 731-0153, Japan

**Keywords:** antibacterial peptide, cathepsin B, cathepsin L, human β-defensins, interleukin-6, lipopolysaccharide, microglia, NF-κB p65, nitric oxide, *Porphyromonas gingivalis*

## Abstract

Recently, the effects of antibacterial peptides are suggested to have therapeutic potential in Alzheimer’s disease. Furthermore, systemic treatment of *Porphyromonas gingivalis* (*Pg*) lipopolysaccharide (LPS) induced Alzheimer’s disease-like neuropathological changes in middle-aged mice. Then, we examined whether human β-defensins (hBDs), antimicrobial peptides produced by the oral mucosa and salivary glands, can suppress *Pg* LPS-induced oxidative and inflammatory responses by microglia. hBD3 (1 μM) significantly suppressed *Pg* LPS-induced production of nitric oxide and interleukin-6 (IL-6) by MG6 cells, a mouse microglial cell line. hBD3 (1 μM) also significantly inhibited *Pg* LPS-induced expression of IL-6 by HMC3 cells, a human microglial cell line. In contrast, neither hBD1, hBD2 nor hBD4 failed to inhibit their productions. Furthermore, hBD3 suppressed *Pg* LPS-induced p65 nuclear translocation through the IκBα degradation. *Pg* LPS-induced expression of IL-6 was significantly suppressed by E64d, a cysteine protease inhibitor, and CA-074Me, a known specific inhibitor for cathepsin B, but not by pepstatin A, an aspartic protease inhibitor. Interestingly, hBD3 significantly inhibited enzymatic activities of recombinant human cathepsins B and L, lysosomal cysteine proteases, and their intracellular activities in MG6 cells. Therefore, hBD3 suppressed oxidative and inflammatory responses of microglia through the inhibition of cathepsins B and L, which enzymatic activities are necessary for the NF-κB activation.

## 1. Introduction

Neuroinflammation is a reaction of the host to infectious or sterile tissue damage, and has the physiological purpose of restoring tissue homeostasis. On the other hand, uncontrolled or unresolved chronic neuroinflammation can lead to neuronal damage. It is well known that chronic neuroinflammation is associated with brain pathologies including Alzheimer’s disease (AD). However, there is limited knowledge regarding the mechanism by which acute neuroinflammation turns into a chronic one. Recently, much attention has been paid to the role of microbial infection in the pathogenesis of sporadic AD [1]. Periodontitis is a common oral chronic multi-bacterial infection which results in a persistent bacterial and inflammatory load in the body. An increasing number of clinical studies have demonstrated the impact of periodontitis on AD, and recent experimental studies clarified the route of transduction of inflammatory signals from periodontitis to the brain. *Porphyromonas gingivalis* (*Pg*), a major pathogen of chronic periodontitis, has virulence factors including lipopolysaccharide (LPS), cysteine proteases called gingipains and outer membrane vesicles [1,2,3,4]. Recently, LPS and gingipains, two major virulence factors of *Pg*, have been detected in the brain tissue of patients with AD on autopsy [5,6]. Cohort studies showed that periodontitis is associated with an increase in cognitive decline in AD patients [7,8]. Therefore, much attention has been paid to the causal relationship between *Pg* virulence factors and cognitive dysfunction in AD patients [9,10,11]. We have first reported that chronic systemic exposure of *Pg* LPS induced AD-like pathologies, including microglia-mediated neuroinflammation and cognitive decline in middle-aged wild-type, but not cathepsin B (CatB)-deficient mice [12]. Furthermore, neuroinflammation is responsible for cognitive impairment following chronic systemic exposure of *Pg* LPS in young adult mice [13] and periodontitis induced by *Pg* LPS in young adult rats [14].

Besides the ubiquitin-proteasome system, there is accumulating evidence demonstrating that the endosomal-lysosomal system is responsible for delayed degradation of IκBα, an endogenous inhibitor of nuclear factor-κB (NF-κB), at the late phase of oxidative and inflammatory responses by macrophages and microglia [15,16,17,18,19,20]. CatB was suggested to promote the autolysosomal degradation of IκBα and subsequent NF-κB nuclear translocation following hypoxic-ischemic brain injury of neonatal mice [18]. These observations suggest that *Pg* LPS has a significant influence over AD pathologies through oxidative and inflammatory responses mediated by activated microglia in a lysosome-dependent manner.

The emerging role of microbes and innate immune pathways in AD pathology suggests that antimicrobial peptides may be considered for early therapeutic interventions in future clinical trials [21,22]. Human β-defensins (hBDs) are cationic and small antimicrobial peptides produced by the oral mucosa and salivary glands. hBD1 and hBD2 are also expressed by microglia and astrocytes of both mouse and human brains [23,24,25]. With the exception of constitutively expressed hBD1, inducible hBD2 and hBD3 are generally up regulated by an inflammatory environment [25]. hBD1 is constitutively expressed in the choroid plexus, which acts as a primary immuno surveillance and defense mechanism against invading pathogens and systemic diseases [26]. Furthermore, the expression of hBD1 is increased within granulovacuolar degeneration structures localized in the cytoplasm of hippocampal pyramidal neurons and astrocytes of AD brain compared with the control brain [27]. A higher level of hBD1 was also seen in the choroid plexus of AD brain in comparison with an age-matched control brain. Moreover, AD patients show higher copy numbers of polymorphism of the DEFB4 gene that encodes for hBD2 and influences the production of hBD2, thus explaining the increased levels of hBD2 reported in the serum and cerebrospinal fluid of AD patients [28]. Human cultured astrocytes have been shown to express hBD2 following treatment with LPS and cytokines [23]. hBD3 is a potent antimicrobial peptide and exhibits immunosuppressive and anti-inflammatory effects on LPS-stimulated macrophages with relatively low concentrations [29,30]. These findings suggest that increased expression of hBDs may ameliorate the pathological progression of AD through regulation of activated microglia.

It remains, however, unclear whether hBDs are able to suppress oxidative and inflammatory responses induced by *Pg* LPS-stimulated microglia. In this study, we have thus sought to elucidate possible inhibitory effects of hBDs on oxidative and inflammatory responses induced by *Pg* LPS-stimulated microglia.

## 2. Results

### 2.1. Effects of hBDs on Cell Viability of MG6

**Figure 1:**
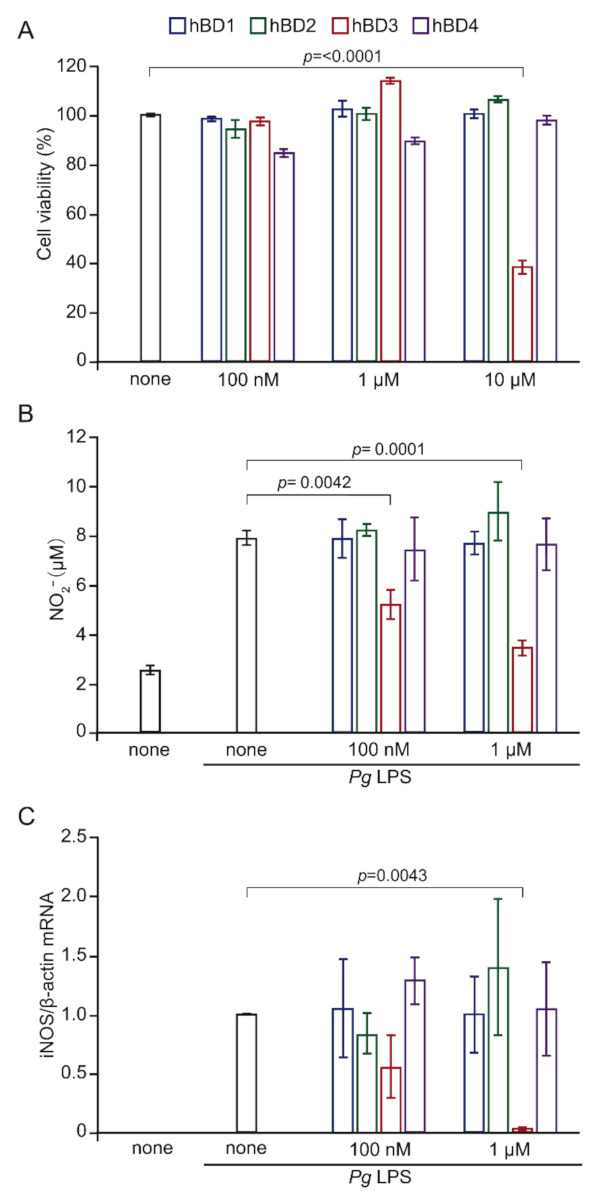
Effect of hBDs on cell viability, NO production and iNOS expression in MG6 cells after treatment of *Pg* LPS. (**A**) Cell viability of MG6 cells was evaluated using CCK-8 assay at 26 h after treatment with hBDs. MG6 cells were incubated with hBD1, 2, 3 and 4 with the concentrations ranging from 100 nM to 10 μM. (**B**) the mean level of nitrite, the major NO metabolite, was measured by the Griess assay. MG6 cells were pre-treated with hBDs at the indicated concentrations (100 nM and 1 μM) for 2 h and then treated with *Pg* LPS (10 μg/mL) for 24 h. **C**. The mean mRNA expression level of iNOS was determined by qRT-PCR analysis and β-actin mRNA served as the internal control for the normalization. MG6 cells were pre-treated with hBDs at the indicated concentrations (100 nM and 1 μM) for 2 h and then treated with *Pg* LPS (10 μg/mL) for 6 h. (**A**–**C**) The data are presented as the mean ± SE of three independent experiments, and *p* values were calculated using a one-way ANOVA with a post-hoc Tukey’s test. A value of *p* < 0.05 was considered to indicate statistical significance.

We first examined effects of hBDs on the cell viability of MG6 cells using the cell counting kit; 8 at 26 h after treatment with hBDs with the concentration ranging from 100 nM to 10 μM. hBD1, 2 and 4 with the concentration up to 10 μM had no significant toxic effect on MG6 cells. On the other hand, hBD3 did not exhibit a significant toxic effect on MG6 cells up to 1 μM, but 10 μM hBD3 significantly reduced the cell viability (Figure 1A).

### 2.2. Effects of hBDs on Pg LPS-Induced Nitric Oxide (NO) Production and Inducible NO Synthase (iNOS) Expression by Microglia

To address whether hBDs could reduce *Pg* LPS-induced NO production by microglia, we next utilized a Griess assay to determine levels of nitrite, the major NO metabolite, in MG6 cells. hBD3 with the concentration of 100 nM and 1 μM significantly reduced nitrite production in MG6 cells after treatment with *Pg* LPS (10 μg/mL) for 24 h (Figure 1B). In contrast, neither hBD1, hBD2 nor hBD4 with the concentration up to 1 μM showed significant effect on the mean level of nitrite produced by MG6 cells after treatment with *Pg* LPS. The mean level of *Pg* LPS-induced iNOS mRNA was also significantly reduced by hBD3 (1 μM), but not by either hBD1, hBD3 or hBD4 (Figure 1C).

### 2.3. Effects of hBDs on Pg LPS-Induced Interleukin-6 (IL-6) mRNA and Protein by Microglia

**Figure 2:**
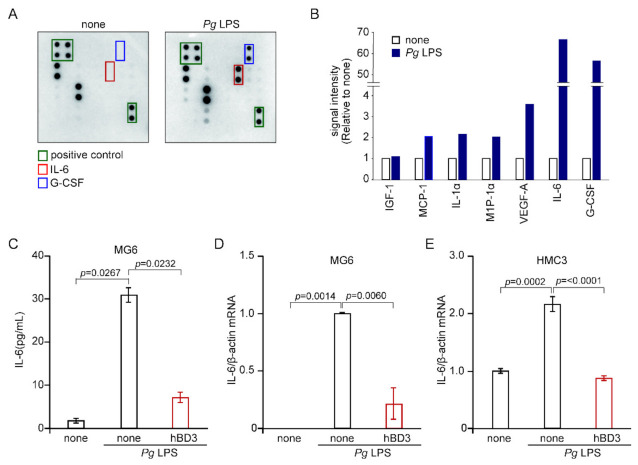
Effects of hBDs on the cytokine expression by microglia after exposure to *Pg* LPS. (**A**) Cytokines secreted from MG6 cells after exposure to *Pg* LPS were detected by cytokine antibody array. Each cytokine is represented by duplicate spots in the locations shown. (**B**) Densitometric analyses of cytokine spots shown in A. Each column represents fold change of optical intensity for average of two spots detected. (**C**) The mean protein level of IL-6 secreted in the culture medium of MG6 cells was determined by ELISA. MG6 cells were pre-treated with hBD3 (1 μM) for 2 h and then treated with *Pg* LPS (10 μg/mL) for 6 h. (**D**) The mean mRNA expression level of IL-6 in MG6 cells was determined by qRT-PCR analysis and β-actin mRNA served as the internal control for the normalization. MG6 cells were pre-treated with hBD3 (1 μM) for 2 h and then treated with *Pg* LPS (10 μg/mL) for 6 h. (**E**) The mean mRNA expression level of IL-6 in HMC3 cells was determined by qRT-PCR analysis and β-actin mRNA served as the internal control for the normalization. HMC3 cells were pre-treated with hBD3 (1 μM) for 2 h and then treated with *Pg* LPS (30 μg/mL) for 3 h. (**C**–**E**) The data are presented as the mean ± SE of three independent experiments, and *p* values were calculated using a one-way ANOVA with a post-hoc Tukey’s test. A value of *p* < 0.05 was considered to indicate statistical significance.

To determine cytokines and chemokines induced by stimulation with *Pg* LPS, we examined the cytokine expression in MG6 cells by using a cytokine antibody array. Untreated MG6 cells (none, distilled water only) were found constitutively to express relatively high levels of insulin growth factor-1 (IGF-1) and macrophage inflammatory protein-1α (MIP-1α), while all of the other cytokines and chemokines were undetectable. Both IL-6 and granulocyte-colony stimulating factor (G-CSF) were most intensively expressed in MG6 cells following stimulation with *Pg* LPS (10 μg/mL) for 24 h (Figure 2A,B). It was also noted that secretion levels of monocyte chemoattractant protein-1 (MCP-1), IL-1α, MIP-1α and vascular endothelial growth factor-A (VEGF-A) were also markedly increased following treatment with *Pg* LPS.

IL-6 is a pleiotropic cytokine that regulates multiple biological processes, including the development of the nervous and hematopoietic systems, acute-phase responses, inflammation, and immune responses [31]. On the other hand, G-CSF is essential for the protective inflammatory response and for maintaining the balance between anti- and pro inflammatory reactions in an inflammatory condition [32]. We then focused on the *Pg* LPS-induced expression of IL-6. Effects of hBD3 on the *Pg* LPS-induced IL-6 expression in MG6 cells were examined at both the protein and transcriptional levels using enzyme-linked immunosorbent assay (ELISA) and quantitative real-time polymerase chain reaction (qRT-PCR), respectively. We examined thee effects of only hBD3 on the expression of IL-6, because the production of NO and IL-6 in microglia uses common signaling pathways. hBD3 (1 μM) significantly inhibited *Pg* LPS-induced expression of IL-6 in MG6 cells at both protein (Figure 2C) and mRNA (Figure 2D) levels. To further generalize the above observations, we used human embryonic microglia clone 3 (HMC3). The responsiveness of HMC3 cells to stimulation with *Pg* LPS (10 μg/mL) are relatively weak when compared with that of MG6 cells. *Pg* LPS (10 μg/mL) could induce expression of IL-6 mRNA in HMC3 cells, but there was a significant variation in its effectiveness. We then used *Pg* LPS with the concentration of 30 μg/mL. *Pg* LPS (30 μg/mL for 3 h) induced a significant increase in the expression of IL-6 mRNA in HMC3 cells (Figure 2E). hBD3 (1 μM) significantly inhibited *Pg* LPS-induced mRNA expression of IL-6 in HMC3 cells (Figure 2E).

### 2.4. Effects of hBD3 on Pg LPS-Induced Nuclear Translocation of p65 and Proteolytic Degradation of IκBα

**Figure 3:**
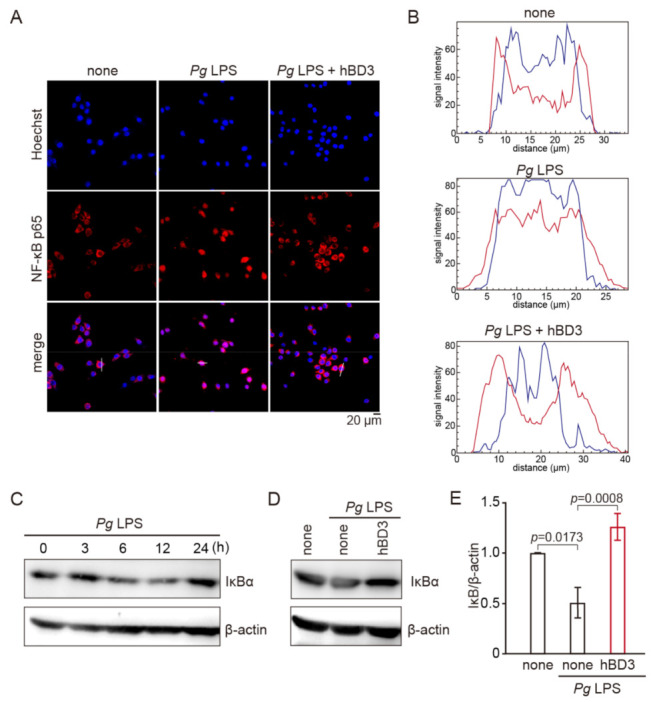
Effects of hBD3 on the *Pg* LPS-induced nuclear translocation of NF-κB p65 and degradation of IκBα. (**A**) Immunofluorescence CLMS images indicating the nuclear translocation of p65 (red) in MG6 cells with Hoechst-stained nuclei (blue) at 3 h after stimulation with *Pg* LPS (10 μg/mL) in the presence or absence of hBD3 (1 μM). (**B**) The typical cells were analyzed by line plot profile to show the cytosol and nuclear location of p65. (**C**) The protein expression of IκBα in MG6 cells after stimulation with *Pg* LPS (10 μg/mL) at the indicated time. (**D**) The protein expression of IκBα in MG6 cells after stimulation with *Pg* LPS (10 μg/mL) for 6 h in the presence or absence of hBD3 (1 μM). (**E**) The mean intensity of IκBα, which were detected by the immunoblots shown in (**D**), were measured and normalized against the signal of β-actin. They are shown here relative to the values in untreated cells. The data are presented as the mean ± SE of five independent experiments from MG6 cells after stimulation with *Pg* LPS for 6–24 h in the presence or absence of hBD3, and *p* values were calculated using a one-way ANOVA with a post-hoc Tukey’s test. A value of *p* < 0.05 was considered to indicate statistical significance.

The promoter regions of both iNOS and IL-6 genes have a putative NF-κB binding site [33,34]. Therefore, abrogation of NF-κB activation is one of the plausible mechanisms underlying the inhibitory effect of hBD3 on the *Pg* LPS-induced NO and IL-6 production in MG6 cells. We thus examined effects of hBD3 on the *Pg* LPS-induced p65 nuclear translocation and degradation of IκBα, an endogenous inhibitor of NF-κB. hBD3 (1 μM) significantly inhibited the *Pg* LPS-induced p65 nuclear translocation in MG6 cells (Figure 3A,B). Furthermore, the mean level of IκBα was significantly decreased in MG6 cells after treatment with *Pg* LPS (10 μg/mL) at 6–12 h (Figure 3C). hBD3 (1 μM) significantly inhibited *Pg* LPS-induced delayed reduction of IκBα (Figure 3D,E).

### 2.5. Inhibitory Effects of hBD3 on Activities of Human CatB and Cathepsin L (CatL)

**Figure 4:**
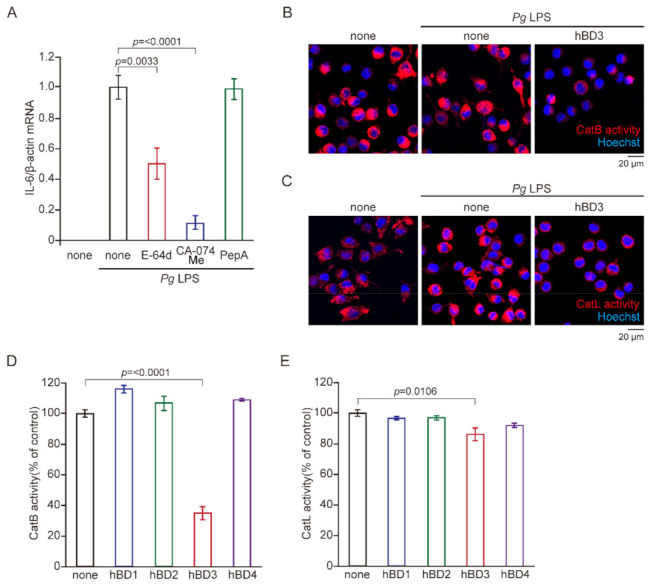
Effects of hBD3 on the enzymatic activity of human CatB and CatL. (**A**) The mean level of *Pg* LPS-induced IL-6 mRNA was significantly reduced by E64d (50 μM) and CA-074Me (30 μM), but not by pepstatin A (30 μM). (**B**,**C**) CLSM images of z-Arg-Arg-cresyl violet (**B**) and z-Phe-Arg-cresyl violet (**C**) in non-treated and *Pg* LPS-treated MG6 cells in the absence and presence of hBD3 (1 μM). (**D**,**E**) The mean enzymatic activity levels of human CatB (**D**) and human CatL (**E**) were measured by measured by AFC based peptide substrates to release AFC. (**A**,**D**,**E**) The data are presented as the mean ± SE of three independent experiments, and *p* values were calculated using a one-way ANOVA with a post-hoc Tukey’s test. A value of *p* < 0.05 was considered to indicate statistical significance.

We previously reported that CatB played a critical role in AD-like pathologies, including microglia-mediated neuroinflammation following a chronic systemic exposure of *Pg* LPS [12]. Furthermore, growing evidence suggests that inflammatory and oxidative responses of macrophages/microglia at the late phase depends on the lysosomal proteolytic machinery [15,16,17,18,19,20]. We thus examined the effects of lysosomal protease inhibitors on the *Pg* LPS-induced expression of IL-6 mRNA. *Pg* LPS-induced expression of IL-6 mRNA in MG6 cells was significantly inhibited by E64d (50 μM), a broad cysteine protease inhibitor, and CA-074Me (30 μM), a known specific inhibitor for CatB, but not by pepstatin A (30 μM), an aspartic protease inhibitor (Figure 4A).

Therefore, possible inhibitory effects of hBD3 on the enzymatic activities of CatB and CatL, typical cysteine cathepsins, were examined by use of cell-permeable, fluorescently labeled substrates, z-Arg-Arg-cresyl violet and z-Phe-Arg-cresyl violet, respectively. The fluorescent cresyl violet group of which is designed to be dequenched upon the cleavage of dipeptides by CatB or CatL. The enzymatic activities of CatB and CatL, visualized as punctuate bright signals in MG6 cells, were increased after stimulation with *Pg* LPS (10 μg/mL). hBD3 (1 μM) markedly reduced the increased fluorescent signals of both CatB and CatL after treatment with *Pg* LPS (Figure 4B,C), suggesting that hBD3 penetrated into MG6 cells to inhibit their enzymatic activities in the lysosomes.

To quantify the inhibitory effects of hBD3 on activities of CatB and CatL, we examined effects of hBDs on the enzymatic activities of recombinant human CatB and CatL using the cleaved synthetic AFC based peptide substrates, Ac-Arg-Arg-AFC and Ac-Phe-Arg-AFC, respectively. hBD3 (1 μM) significantly inhibited enzymatic activities of recombinant human CatB and CatL (Figure 4D,E). In contrast, either hBD1, 2 or 4 showed no significant effect on their enzymatic activities, suggesting that their lack of inhibitory effect on the CatB and CatL activities is responsible for their inability of inhibiting *Pg* LPS-induced oxidative and inflammatory responses.

## *3.* Discussion

In this study, we sought to determine whether hBDs could reduce *Pg* LPS-induced oxidative and inflammatory responses in microglia. Our observations indicate that hBD3 with a relatively low concentration (i.e., 1 μM) significantly suppressed *Pg* LPS-induced production of NO and iNOS by MG6 cells. Furthermore, hBD3 also significantly suppressed *Pg* LPS-induced expression of IL-6 by MG6 cells in both mRNA and protein levels. The inhibitory effect of hBD3 on *Pg* LPS-induced mRNA expression of IL-6 was also observed in HMC3 cells. In contrast, either hBD1, hBD2 or hBD4 failed to inhibit the production of NO and IL-6 by MG6 cells.

Chronic systemic exposure of *Pg* LPS induces AD-like pathology, including microglia-mediated neuroinflammation and cognitive decline in both mice and rats [12,13,14]. There is increasing evidence of lysosome-related degradation of IκBα, an endogenous inhibitor of NF-κB, at the late stage of inflammation [15,16,17,18,19,20]. Several studies have revealed that hBD3 inhibits *E. coli* LPS-induced inflammatory responses of the macrophage cell line RAW 264.7 cells by down regulation of NF-κB and inflammatory gene transcription [35], or directly suppressing the degradation of phosphorylated IκBα [36]. In this study, hBD3 significantly suppressed the *Pg* LPS-induced p65 nuclear translocation. It has been reported that standard *Pg* LPS, which contains other bacterial components including lipoproteins, could activate the NF-κB/STAT3 signaling pathways in BV-2 microglial cells through Toll-like receptor 2/4 (TLR2/4) [37,38]. Therefore, it is considered that hBD3 inhibits *Pg* LPS-induced p65 nuclear translocation through the IκBα degradation and subsequent NF-κB activation at the late stage of neuroinflammation, which may drive delayed and/or chronic state.

hBD3 is highly membrane-permeable, because it has a cationic amino acid sequence in the C-terminus and a hydrophobic amino acid sequence in the N-terminus (Supplementary Figure S1) [36,39]. Therefore, the potent cell-penetrating activity of hBD3 is partly responsible for the antioxidant and anti-inflammatory effects in microglia. In this study, *Pg* LPS-induced expression of IL-6 by MG6 cells was significantly inhibited by E64d, a cysteine cathepsin inhibitor, and CA-074Me, a known specific inhibitor for CatB, but not by pepstatin A, a specific aspartic protease inhibitor. Moreover, it has been reported that hBD2 and hBD3 could be substrates for cysteine cathepsins such as CatB and CatL [40]. These observations further prompted us to examine the effects of hBD3 on the enzymatic activities of cysteine cathepsins. As expected, hBD3 (1 μM) significantly inhibited the enzymatic activities of recombinant human CatB and CatL, probably through the suicide substrate-based inhibition mechanism. hBD3 (1 μM) also inhibited enzymatic activities of both CatB and CatL in MG6 cells by use of cell-permeable fluorescently labeled substrates of CatB and CatL (Figure 4B,C). The punctate fluorescent signals, which indicate enzymatic activity in the endosomes/lysosomes, were markedly reduced by treatment with hBD3, suggesting that hBD3 can penetrate into the endosomes/lysosomes. In contrast, either hBD1, 2 or 4 showed no significant effects on their activities. On the basis of these observations, their lack of an inhibitory effect on the CatB and CatL activities, rather than their weak membrane permeability, is responsible for the inability of inhibiting *Pg* LPS-induced oxidative and inflammatory responses. Therefore, it may be concluded that hBD3 suppresses the *Pg LPS*-induced oxidative and inflammatory responses in microglia through inhibiting CatB and CatL, which enzymatic activities are necessary for degradation of IκBα at the late stage of inflammation. The present results are consistent with our previous findings. Both the genetic deficiency of CatB and its pharmacological inhibition by CA-074Me ameliorated microglia-mediated neuroinflammation and cognitive impairment of *Pg* LPS-treated middle-aged mice [12]. Furthermore, CA-074Me significantly inhibited the *Pg* infection-induced Aβ peptide production by macrophages [41]. It is worthwhile to mention that CA-074Me with relatively high concentrations inhibits not only CatB, but also other cysteine cathepsins such as CatL in mammalian cells [42,43].

In the brain, astrocytes, epithelium of the choroid plexus and meningothelial cells express and likely secrete immuno-modulatory antimicrobial peptides, including hBD1 and hBD2, that could influence inflammation within the brain [23,26]. It has been reported that salivary lactoferrin is transferred into the brain by absorption from the sublingual mucosa, in which the favorable effects of salivary lactoferrin on brain will be expected via the sublingual mucosa [44]. hBDs including hBD3 are produced by the oral mucosa and salivary glands. Therefore, it is interesting to speculate that hBD3 can be also transferred into the brain through the sublingual route. The relationship between salivary glands and brain function might provide new insight into potential therapeutic approaches for brain neurological disorders [45]. Further study will be necessary to elucidate the absorption and transportation of salivary hBD3 into the brain.

## 4. Materials and Methods

### 4.1. Reagents

hBD1-4, E64d, CA-074Me and pepstatin A were purchase from Peptide Institute Inc. (Osaka, Japan). Standard *Pg* LPS was purchased from Invivogen (San Diego, CA, USA).

### 4.2. Cell Culture

The c-myc-immortalized mouse microglial cell line, MG6 (RIKEN Cell Bank), was maintained at 37 °C in DMEM containing 10% heat-inactivated fetal bovine serum (FBS, ICN Biomedicals) supplemented with 3.5 mg/mL glucose, 100 μM β-mercaptoethanol, 10 μg/mL insulin, 100 μg/mL streptomycin, and 100 U/mL penicillin (ThermoFischer Scientific, Walthan, MA, USA). HMC3 (CRL-3304) cells obtained from American Type Culture Collection (ATCC, Manassas, VA, USA) were maintained at 37 °C in DMEM containing 10% heat-inactivated FBS supplemented with 2 mg/mL glucose, 2 mM glutamine, 100 units/mL penicillin, and 100 μg/mL streptomycin.

### 4.3. Assay for Cell Survival

Cell viability was measured by WST-8 conversion to water-soluble formazan by mitochondrial dehydrogenase (Cell Counting Kit-8, Dojindo, Japan) following the protocol provided by the manufacturer. Briefly, WST-8 was added to MG6 cells in 96-well plates (3 × 10^4^ cells/well) and incubated at 37 °C for 1 h. The optical density was read at a wavelength of 450 nm with a microplate reader.

### 4.4. Assay of NO Production

MG6 cells cultured in a 96-well plate (3 × 10^4^ cells/well) were pre-treated with hBD1, 2, 3, and 4 at 100 nM and 1 μM for 2 h and then exposed to *Pg* LPS (10 μg/mL) for 24 h. The supernatant was transferred to a new 96-well plate with Griess reagent (Griess Reagent Kit; Dojindo, Kumamoto, Japan) and incubated at room temperature for 15 min. The amount of nitrite, the major NO metabolite, in the cytosol was measured spectrophotometrically using a microplate reader at a wavelength of 540 nm.

### 4.5. qRT-PCR

Total RNA was extracted using TRIzol regent (ThermoFischer Scientific) according to the manufacturer’s instructions. 1–2 μg of total RNA was used for cDNA synthesis using the ReverTra Ace (TOYOBO, Osaka, Japan). After a denaturation step at 95 °C for 1 min, 45 cycles of denaturation at 95 °C for 15 s, annealing at 61 °C (iNOS (mouse)), 57 °C (IL-6 (mouse)), 55 °C (β-actin (mouse)), or 52°C (IL-6 (human) and β-actin (human)) for 15 s and extension at 72 °C for 29 s (iNOS (mouse)), 20 s (IL-6 (mouse)), or 1 min (β-actin (mouse), IL-6 (human), β-actin (human)) were carried out to amplify each cDNA. The cDNA was amplified in triplicate using a Thunderbird next SYBR qPCR mix (TOYOBO) with Real-Time PCR System TP800 (TaKaRa-bio, Shiga, Japan) or TP990 (TaKaRa-bio). The sequences of primer pairs were described as follows: iNOS (mouse): 5′-CTCGGAACTGTAGCACAGCAC-3′ and 5′-AAGACCAGAGGCAGCACATCAA-3′; IL-6 (mouse): 5′-TCTTGGGACTGATGCTGGTG-3′ and 5′-GCA CAA CTC TTT TCT CATTTCC-3′; β-actin (mouse): 5′-GGCATTGTGATGGACTCCG-3′ and 5′-GCT GGA AGGTGGACAGTGA-3′; IL-6 (human): 5′-GAACTCCTTCTCCACAGCG-3′ and 5′-TTTTCTGCCAGTGAATCTTT-3′; β-actin (human): 5′-CAT CTCTTGCTCGAAGT CCA-3′ and 5′-ATCATGTTTGAGACCTT CAACA-3′. For data normalization, an endogenous control (β-actin) was assessed to control for the cDNA input, and the relative units were calculated by a calibration curve method. All qRT-PCR experiments were repeated three times, and the results are presented as the means of the ratios ± SE.

### 4.6. Cytokine Antibody Array

MG6 cells were exposed to *Pg* LPS (10 μg/mL) at 37 °C for 24 h. Culture supernatant were then analyzed with cytokine antibody array by using the C-Series Mouse Neuro Discovery Array C1 (RayBiotech, Norcross, GA, USA) according to the manufacturer’s instructions. Signal intensities were detected with LAS-4000 (Fujifilm, Tokyo, Japan) and analyzed with Image Lab 6.0.1 software program (Bio-Rad, Hercules, CA, USA). Biotin-conjugated immunoglobulin G served as a positive control at six spots, where it was used to identify membrane orientation and to normalize the results from different membranes that were being compared. Following, 23 molecules containing cytokines and chemokines were detected and semi quantified on membrane arrays containing different cytokine antibodies: Fas ligand, fractalkine, G-CSF, interferon-γ, IGF-1, IL-10, IL-1α, IL-1β, IL-4, IL-6, keratinocyte chemoattractant, LPS-induced CXC chemokine, MCP-1, MIP-1α, macrophage-colony stimulating factor, matrix metaloprotease-2, matrix metaloprotease-3, receptor for advanced glycation end products, thymus and activation-regulated chemokine, stromal cell-derived factor-1α, transforming growth factor-β, tumor necrosis factor-α, VEGF-A.

### 4.7. ELISA

The amounts of secreted IL-6 from MG6 cells were measured by ELISA (RayBiotech Inc., Norcross, GA, USA) following the protocol provided by the manufacturer. The absorbance at 450 nm was determined using a microplate reader.

### 4.8. Immunoblotting Analyses

MG6 cells were seeded on a petri dish at a density of 7.5–10 × 10^5^ cells/dish for 1–2 days. After treatment with *Pg* LPS for 3, 6, 12, 24 h, the cells were then lysed with RIPA buffer, consisting of 10 mM Tris-HCl, pH 7.5, 1% (*v*/*v*) NP-40, 0.1% (*w*/*v*) sodium deoxycholate, 0.1% (*w*/*v*) SDS, 150 mM NaCl, 1 mM EDTA, protease inhibitor cocktail (Nacalai Tesque, Kyoto, Japan), and then cell lysates were subjected to 10% (*w*/*v*) sodium dodecyl sulfate-polyacrylamide gel electrophoresis. The proteins on the SDS–polyacrylamide gels were then transferred to nitrocellulose membranes. After blocking with Blocking one (Nacalai Tesque), the membranes were incubated with a rabbit anti-IκBα (Abcam, Cambridge, UK) and rabbit-anti-β-actin (GENETEX, Irvine, CA, USA) at 4 °C overnight. After being washed, the membranes were incubated with horseradish peroxidase (HRP)-labeled anti-rabbit IgG antibodies (GE Healthcare, Tokyo, Japan) for 1 h at room temperature. Subsequently, the membrane-bound HRP-labeled antibodies were detected using an Amasham ECL western blotting detection reagent and analysis system (GE Healthcare) with an imaging analyzer (LAS-4000, Fujifilm, Tokyo, Japan). Signal intensities were determined using the Image Lab 6.0.1 software program (Bio-Rad, Hercules, CA, USA).

### 4.9. Enzymatic Activity Assay of CatB and CatL

The enzymatic activities of CatB or CatL in the absence and presence of hBDs were measured by the cleaved synthetic 7-amino-4-trifluoromethylcoumarin (AFC) based peptide substrates, Ac-Arg-Arg-AFC and Ac-Phe-Arg-AFC, respectively (BioVision, Milpitas, CA, USA). The fluorescence intensities of AFC were measured by a microplate reader at an excitation wavelength of 400 nm and an emission wavelength of 505 nm.

### 4.10. Fluorescence Imaging of Enzymatic Activities of CatB and CatL

MG6 microglia were stained with the cell-permeable fluorescently labeled CatB substrate z-Arg-Arg-cresyl violet or CatL substrate z-Phe-Arg-cresyl violet according to the manufacturer’s instructions (cv-CatB detection kit and cv-CatL detection kit; Enzo Life Sciences, Inc., Farmingdale, NY, USA). Chamber slides containing the stained live cells were then mounted in PBS. Fluorescent images were taken using a confocal laser-scanning microscope (CLSM, FV1000, Olympus, Tokyo, Japan).

### 4.11. Immunostaining

MG6 cells were treated with *Pg* LPS (10 μg/mL) for 3 h in the absence and presence of hBD3 (1 μM) and were fixed with 4% paraformaldeyte. They were then incubated with rabbit anti-NF-κB p65 IgG (Abcam). After washing with PBS, cells were incubated with donkey anti-rabbit Alexa 555 (ThermoFisher Scientific, Waltham, MA, USA), then incubated with Hoechst and mounted in Vectashield anti fading medium (Vector Laboratories, Newark, CA, USA). Fluorescent images were taken using a confocal laser-scanning microscope (FV1000). The line plot profile was analyzed using Image J.

### 4.12. Statistical Analysis

The data are represented as the mean ± standard error (SE). Statistical analyses of the results were performed with one-way analysis of variance (ANOVA) with post hoc Tukey’s test using the GraphPad Prism8 (GraphPad Software, Inc., San Diego, CA, USA) software package. *p* < 0.05 was considered to indicate a statistically significant difference.

## 5. Conclusions

hBD3 inhibited *Pg* LPS-induced oxidative and inflammatory responses in microglia through a direct inhibition of CatB and CatL. Both CatB and CatL might be involved in degradation of IκBα, resulting in a delayed but long-lasting activation of NF-κB pathways.

## Data Availability

Not applicable.

## Supplementary Materials

The supporting information can be downloaded at: https://www.mdpi.com/article/10.3390/ijms232315099/s1.

## Abbreviations

AFC: 7-amino-4-trifluoro-methylcpumarin; AD: Alzheimer’s disease; ANOVA: one-way analysis of variance; CatB: cathepsin B; CatL: cathepsin L; CLMS: confocal laser-scanning microscope; ELISA: enzyme-linked immunosorbent assay; G-CSF: granulocyte growth factor; hBDs: human β-defensins; HMC3: human embryonic microglia clone 3; IGF: insulin growth factor; IL-6: interleukin-6; iNOS: inducible nitric oxide synthase; LPS: lipopolysaccharide; MCP-1: monocyte chemoattractant protein-1; MIP-1α: macrophage inflammatory protein-1α; NF-κB: nuclear factor-κB; NO: nitric oxide; *Pg*: *Porphyromonas gingivalis*; qRT-PCR: quantitative real-time polymerase chain reaction; SE: standard error; TLR: Toll-like receptor; VEGF-A: vascular endothelial growth factor-A.
