# Supplementary material for: Human β-Defensin 3 Inhibits Porphyromonas Gingivalis Lipopolysaccharide-Induced Oxidative and Inflammatory Responses of Microglia by Suppression of Cathepsins B and L
Source: Int J Mol Sci. 2022 Dec 1;23(23):15099. doi: 10.3390/ijms232315099 (PMC9738813; doi:10.3390/ijms232315099)
Supplement: Supplementary file 1 [file ijms-23-15099-s001.zip › ijms-2020754-supplementary.pdf]

hBD1： DHYNCVSSGGQCLYSACPIFTKIQGTCYRGKAKCCK
hBD2： GIGDPVTCLKSGAICHPVFCPRRYKQIGTCGLPGTKCCKKP
hBD3： GIINTLQKYYCRVRGGRCAVLSCLPKEEQIGKCSTRGRKCCRRKK
hBD4： EFELDRICGYGTARCRKKCRSQEYRIGRCPNTYACCLRKWDESLLNRTKP

Supplementary Figure S1. Amino acid sequences of hBD1-4. Cationic amino acids (R, L, H) are colored with red. Hydrophobic amino acids (G, A, V, L, I, P, F) are colored with blue. Cysteine residues are colored with brown.
